# ParaDB: A manually curated database containing genomic annotation for the human pathogenic fungi *Paracoccidioides* spp.

**DOI:** 10.1371/journal.pntd.0007576

**Published:** 2019-07-15

**Authors:** David Aciole Barbosa, Fabiano Bezerra Menegidio, Valquíria Campos Alencar, Rafael S. Gonçalves, Juliana de Fátima Santos Silva, Renata Ozelami Vilas Boas, Yara Natércia Lima Faustino de Maria, Daniela Leite Jabes, Regina Costa de Oliveira, Luiz R. Nunes

**Affiliations:** 1 Núcleo Integrado de Biotecnologia, Universidade de Mogi das Cruzes (UMC), Mogi das Cruzes, São Paulo, Brazil; 2 Centro de Ciências Naturais e Humanas, Universidade Federal do ABC (UFABC), São Bernardo do Campo, São Paulo, Brazil; Universidad de Antioquia, COLOMBIA

## Abstract

**Background:**

The genus *Paracoccidioides* consists of thermodymorphic fungi responsible for Paracoccidioidomycosis (PCM), a systemic mycosis that has been registered to affect ~10 million people in Latin America. Biogeographical data subdivided the genus *Paracoccidioides* in five divergent subgroups, which have been recently classified as different species. Genomic sequencing of five *Paracoccidioides* isolates, representing each of these subgroups/species provided an important framework for the development of post-genomic studies with these fungi. However, functional annotations of these genomes have not been submitted to manual curation and, as a result, ~60–90% of the *Paracoccidioides* protein-coding genes (depending on isolate/annotation) are currently described as responsible for hypothetical proteins, without any further functional/structural description.

**Principal findings:**

The present work reviews the functional assignment of *Paracoccidioides* genes, reducing the number of hypothetical proteins to ~25–28%. These results were compiled in a relational database called ParaDB, dedicated to the main representatives of *Paracoccidioides* spp. ParaDB can be accessed through a friendly graphical interface, which offers search tools based on keywords or protein/DNA sequences. All data contained in ParaDB can be partially or completely downloaded through spreadsheet, multi-fasta and GFF3-formatted files, which can be subsequently used in a variety of downstream functional analyses. Moreover, the entire ParaDB environment has been configured in a Docker service, which has been submitted to the GitHub repository, ensuring long-term data availability to researchers. This service can be downloaded and used to perform fully functional local installations of the database in alternative computing ecosystems, allowing users to conduct their data mining and analyses in a personal and stable working environment.

**Conclusions:**

These new annotations greatly reduce the number of genes identified solely as hypothetical proteins and are integrated into a dedicated database, providing resources to assist researchers in this field to conduct post-genomic studies with this group of human pathogenic fungi.

## Author summary

The genus *Paracoccidioides* comprises fungi responsible for Paracoccidioidomycosis (PCM), a neglected tropical disease prevalent in South America that has been shown to affect approximately 10 million people and has great medical/social impact, since available treatments are poorly effective, frequently leading to relapses, chronic infections and sequelae. Genomic information available for five reference *Paracoccidioides* isolates could greatly assist researchers in developing new chemotherapeutic approaches against PCM, but usefulness of such data is limited, since ~60–90% of *Paracoccidioides* protein-coding genes (depending on isolate) are described as responsible for hypothetical proteins, without any functional/structural description. Such elevated number of hypothetical proteins is unexpected and probably derives from annotations performed solely by automated computing pipelines. This problem can be minimized by manual curation, when expert reviewers determine the functional designation of each gene, after comparing results derived from several reference databases. This work describes an effort to review the functional assignment of >40,000 genes, annotated across the five *Paracoccidioides* genomes mentioned above, which reduced the number of hypothetical proteins to ~25–28%, contributing to significantly increase quality and usefulness of such genomic information. These data have been compiled in a relational database named ParaDB, constituting an important resource for researchers in the field.

## Introduction

The genus *Paracoccidioides* includes a series of thermodymorphic fungi responsible for causing a neglected tropical disease known as paracoccidioidomycosis (PCM), which represents one of the most prevalent systemic mycoses in Latin America [1]. In fact, approximately 10 million people have been estimated to be infected by these fungi, which are distributed in large areas of Brazil, Argentina, Colombia, Venezuela, Ecuador and Paraguay [1,2,3,4,5]. The genus *Paracoccidioides* was originally proposed in 1908, containing a single species, called *P*. *brasiliensis* [6]. Subsequent studies led to the characterization of several isolates, from different geographical regions, that display significant genetic variability, as well as differences in many biological characteristics, such as adaptability to laboratory culture, virulence and the ability to induce different host responses [7]. These isolates were initially distributed into five distinct subgroups (Pl, S1, PS2, PS3 and PS4) that have been unofficially considered cryptic *P*. *brasiliensis* species over the years [8,9]. In 2009, the P1 subgroup was classified as a new species, called *P*. *lutzii*, since its isolates display deeper genetic divergence, when compared with representatives of the other subgroups, which remained classified as members of the *P*. *brasiliensis* species complex [7,10]. More recently, the four subgroups within the *P*. *brasiliensis* complex have also been described as different species: *P*. *brasiliensis* (subgroup S1), *P*. *americana* (subgroup PS2), *P*. *restrepiensis* (subgroup PS3) and *P*. *venezuelensis* (subgroup PS4) [11].

Genomic studies involving *Paracoccidioides* spp. started to be developed in 2003, by large-scale sequencing/characterization of Expressed Sequence Tags (ESTs) obtained from the isolate Pb18, which is the main representative of *P*. *brasiliensis* (S1 subgroup) [12,13]. Subsequently, functional studies, based on the information derived from these EST analyses, demonstrated the potential of genomic approaches to increase our knowledge regarding the genetic bases that determine virulence in these fungi, as well as to provide information that may contribute to the development of new alternatives for the control and treatment of PCM [14,15,16]. These pioneering studies motivated the development of complete genome projects, which led to the characterization of draft genomes of three *Paracoccidioides* isolates: Pb01, Pb03 and Pb18 (representatives of *P*. *lutzii*, *P*. *americana* and *P*. *brasiliensis*, respectively) [17]. This work represented an important milestone to the genetic study of this group of fungi, providing clues that helped us to better understand the evolution of the genus *Paracoccidioides*, as well as a series of genomic characteristics that differentiate some of the abovementioned species/subgroups. However, the sequencing of these three isolates was performed using Sanger technology, generating assemblies with large contig numbers and presenting several regions with low quality consensus sequences, which led to the development of incomplete and inaccurate genomic annotations (v1) for these fungi [17]. Later on, these same isolates were submitted to a new sequencing, using Illumina's NGS platform, in order to produce more complete and precise assemblies [18]. Moreover, a re-annotation analysis performed with such assemblies allowed recovery of a large number of genes that were missed by the original annotation (v1), and this second annotation (v2) was more consistent across the three reference genomes (Pb18, Pb03, and Pb01). Finally, these analyses were extended to contemplate the genomes of additional isolates, representing *P*. *restrepiensis* (isolate PbCnh) and *P*. *venezuelensis* (isolate Pb300), providing reference genomes and annotations for isolates representing all five species/subgroups of the genus *Paracoccidioides* [19].

Currently, genomic data from these five *Paracoccidioides* isolates can be obtained from several generic databases, such as GenBank [20] and Ensembl [21], as well as from some fungal specific databases, such as MycoCosm [22] or FungiDB [23], but the genomic annotations provided through all these repositories are inconsistent and display an unusually large number of protein-coding genes described as responsible for hypothetical proteins. For example, GenBank and RefSeq describe ~62% of all Pb01 genes in association with hypothetical proteins and this proportion is even larger (up to 88%) in the genomes of Pb18, Pb03, Pb300 and PbCnh. A similar situation is observed in other databases, such as Ensembl and FungiDB, which provide the same annotation data found in GenBank/RefSeq. On the other hand, MycoCosm presents an alternative annotation for Pb18, in which a smaller proportion of genes (~68%) is described as associated with hypothetical proteins. However, MycoCosm does not present any information regarding other *Paracoccidioides* isolates (except for Pb03, but these data are based on outdated sequencing information, as they relate to the first version of the Pb03 genome, described by [17]). All these discrepancies, as well as the overall low level of functional gene categorization observed among *Paracoccidioides* isolates may partly derive from the fact that the abovementioned databases have not been submitted to appropriate manual curation, since they are dedicated to providing genomic information for a large number of organisms that may share little genomic similarity or phylogenetic proximity.

Thus, to improve and standardize the current genomic functional annotations of the main *Paracoccidioides* isolates, coding sequences (CDSs) derived from the latest genomic assemblies obtained for Pb18, Pb03, Pb01, PbCnh and Pb300 [18,19] were initially submitted to comparative BLAST analyses against a series of databases, including generic functional databases (InterPro, Pfam and Swiss-Prot) [24,25,26] and fungal-specific, manually-curated databases (*Saccharomyces* Genome Database, *Candida* Genome Database and *Aspergillus* Genome Database) [27,28,29].

Information derived from all these BLAST analyses were compiled in spreadsheets, along with specific Gene Ontology (GO) classifications [30,31]. This metadata was used to develop a manually curated consensus annotation for each of these *Paracoccidioides* genomes. As a result of this process, the number of genes described in association with hypothetical proteins has been reduced to ~25–28%, in all isolates. The information derived from this reannotation effort has been compiled in a publicly available database named ParaDB (available at http://*paracoccidioides*.com) [32], aimed at centralizing up-to-date genomic annotations for the major representatives of the five species/subgroups that compose the genus *Paracoccidioides*. Using a friendly graphical interface, ParaDB allows users to browse and download functional information for any set of genes from any of the abovementioned *Paracoccidioides* genomes. The ParaDB webpage also provides search tools based on keywords or DNA/protein sequence similarity, as well as fully reannotated genome files, in multi-fasta or General Feature (GFF3) formats, which may greatly assist researchers in a variety of large-scale, post-genomic studies with this important group of human pathogenic fungi. Finally, the entire ParaDB environment has been configured in a Docker service [33], which has been submitted to both the GitHub and Open Science Framework repositories, ensuring long-term data availability to researchers. This service can be downloaded and used to perform fully functional local installations of the database in alternative computing ecosystems, allowing users to conduct their data mining and analyses in a personal and stable working environment.

## Methods

### Identification of orthologous genes across the genomes of *Paracoccidioides* spp

Files containing annotated protein coding sequences (CDS genomes) of the *Paracoccidioides* isolates were downloaded from NCBI, using the following accession numbers: Pb18 (RefSeq# GCF_000150735.1), Pb03 (GenBank# GCA_000150475.2), Pb300 (GenBank# GCA_001713645.1), PbCnh (GenBank# GCA_001713695.1), and Pb01 (RefSeq# GCF_000150705.2). The CDSs from these genomes were compared against each other, in order to identify all groups of orthologous genes (OGs) shared by two or more of the isolates, with the aid of the software OrthoFinder [34], using the software´s default parameters. Paralogous genes present within the same OG group were compared by multiple alignment, using Clustal Omega 1.2.4 [35]. The input parameters were set as follow: Output guide tree: false; Output distance matrix: false; Dealign input sequences: false; mBed-like clustering guide tree: true; mBed-like clustering iteration: true; Number of iterations: 0; Maximum guide tree iterations: -1; Maximum HMM iterations: -1. The Nexus-formatted matrix generated by Clustal Omega was then used to estimate genealogical relationships with the aid of Bayesian inference, using Mr. Bayes 3.0 [36]. The analysis involved 1,000,000 iterations, with savings at every 100^th^ tree, 1,100,000 generations, in four heated Monte Carlo Markov chains (MCMCs), with 0.5 annealing temperature, 100 000 MCMC generation burn-in and a 16-category C distribution. A consensus tree was generated after burn-in, using a 50% majority rule, which allowed discrimination between orthologous and co-orthologous genes in the different OG groups. This list of orthologues was then used as a guide to ensure consistent annotation of equivalent genes throughout the five *Paracoccidioides* isolates, during the reannotation process (see below).

Several genes that transcribe non-coding RNAs (ncRNAs) have been identified and annotated in the genomes of Pb01 and Pb18 (the only formal datasets available for ncRNAs in *Paracoccidioides* spp.). Thus, their sequences were downloaded from the Ensembl Fungi database ftp site [21] and orthologues for each of these ncRNA genes were mapped in the genomes of Pb03, PbCnh and Pb300, using Bwa-MEM, version 0.7.17.1 [37], running in a local Galaxy environment [38], using the default software parameters. The resulting BAM alignment files were converted to BED files, with the aid of BAM-to-BED Converter, version 2.27.1 [39,40], also using default software parameters, which facilitated organizing and comparing the predicted ncRNAs across all *Paracoccidioides* spp. isolates. Finally, information regarding these ncRNAs was incorporated into GFF3 files (see below), with the aid of BED-to-GFF Converter [41], version 2.0.0, also using default parameters. All ncRNAs (along with their respective annotations) received identification codes consistent with the ones currently employed to describe Gene_IDs in each *Paracoccidioides* genome, but containing the designation NC (for non-coding) as a suffix. Thus, ncRNAs mapped in the genome of Pb18 received Gene_IDs starting from PADGNC_00001, while ncRNAs for Pb01, Pb03, Pb300 and PbCnh received Gene_IDs starting from PAAGNC_00001, PABGNC_00001, ACO22NC_00001 and GX48NC_00001, respectively. Genes responsible for transcribing additional ncRNAs, including tRNAs and rRNA genes (18S, 28S and 5S rRNAs) had been previously described in the original annotations of the *Paracoccidioides* spp. genomes [18] and sequences for such elements were available from the "rna_from_genomic" fasta files downloaded from GenBank/RefSeq [20]. These genes were also matched to their respective orthologues, using the same procedure described above, but we chose not to change their respective gene IDs (i.e.: they were not labeled with the designation NC), in order to respect their current GenBank/RefSeq IDs.

### Functional reannotation

The overall process employed for reannotating the *Paracoccidioides* genomes is schematically shown in S1 Fig. Initially, all CDSs from Pb18 were individually submitted to comparative BLAST analyses against InterPro, Pfam and Swiss-Prot. Next, these CDSs were BLASTed against the manually-curated fungal databases SGD (*Saccharomyces* Genome Database), CGD (*Candida* Genome Database) and AspGD (*Aspergillus* Genome Database) [27,28,29]. All BLAST analyses employed high stringency criteria, which included as cutoff, an E-value < e^-10^, to identify orthologous genes containing functional descriptions among these databases. Information derived from all these BLAST analyses were compiled in a spreadsheet, along with Gene Ontology (GO) data regarding Pb18 genes, obtained from the Database for Annotation, Visualization and Integrated Discovery (DAVID), version 6.8 [42]. GO data were downloaded through the GO Direct option, in order to reduce the redundancy of terms, typically observed in GO analyses. Next, the metadata regarding each CDS was independently analyzed by three expert reviewers, in order to determine a consensus annotation term (ParaDB Annotation) for each CDS, which should be consistent with all the information derived from DAVID and from the BLAST searches previously performed. In a second round of annotation, a fourth reviewer compared the results of the three independent analyses and determined a final ParaDB Annotation term for each CDS. Finally, all information regarding the ParaDB Annotation, obtained for each Pb18 CDS, was transferred to the orthologues present in any of the *Paracoccidioides* isolates, using the list of orthologous genes (available at http://*paracoccidioides*.com/*paracoccidioides*-orthologous/) as a guide.

Next, all CDSs present in the genome of Pb01, which did not contain an orthologue in Pb18, were submitted to the same analysis procedure described in the previous paragraph. The same process was successively repeated with the remaining CDSs from Pb03, Pb300 and PbCnh, generating thorough and consistent annotations for all *Paracoccidioides* genomes.

In a subsequent annotation step, all CDSs that remained identified as hypothetical proteins were submitted to additional BLAST analyses, using a less stringent cutoff (E-value < e^-5^), essentially as described above. These CDSs were incorporated into the ParaDB database (see below) with a flag (E-value < e^-5^) highlighting the lower stringency criterion used to determine their respective functional annotation.

### Availability of the generated data

The information derived from the reannotation process described above has been compiled in a relational database named ParaDB, aimed at centralizing up-to-date genomic annotations for the major representatives of the five species/subgroups that compose the genus *Paracoccidioides*. ParaDB is available at the URL http://*paracoccidioides*.com and provides users with tabular archives describing each CDS and ncRNA sequences, including their final ParaDB Annotation consensus description, along with information derived from all databases evaluated during the present study. Additional information regarding these elements in all five *Paracoccidioides* isolates can also be downloaded (as nucleotide, or amino acid sequences) through multi-FASTA files, carrying strings that show, for each element, their respective locus_tag ID, protein_product ID and consensus_description (ParaDB Annotation). Updated General Feature Format (GFF3) files for each genome are also available through ParaDB, to assist researchers interested in performing large-scale OMICs analyses with the *Paracoccidioides* spp. genomes. These GFF3 files have been built upon the original GFF3 files available in RefSeq (Pb18 and Pb01) and GenBank (Pb03, Pb300 and PbCnh), by replacing the original GenBank/RefSeq annotations with the respective ParaDB Annotation, with the help of MS Excel. Information regarding the ncRNAs obtained from Ensembl were introduced in the GFF3 files using the BED-to-GFF Converter, as described above.

### Computational structure of ParaDB

The ParaDB environment is based on the Database Management System (DBMS) MySQL and was developed in Docker [33]. Management configuration of the DB was made using Rancher [43], a robust Docker systems management tool, widely used in data center environments and other complex computing ecosystems. The Rancher cluster created to manage ParaDB resources is hosted in a cloud computing environment at CloudatCost [44]. Currently, the environment provides a total of 200 GB of disk space, in solid state drives (SSDs), and 20 GB of random-access memory (RAM). These resources are distributed in 10 virtual CPUs (vCPUs), in a Kubernetes cluster framework [45] (see S2 Fig for details). The ParaDB user interface has been developed in PHP language, using Wordpress [46] and tools to assist in keyword/BLAST searches were implemented with the help of Wordpress plugins and widgets [47].

A Docker service, carrying the Docker virtualization containers necessary to run ParaDB can be downloaded from https://cloud.docker.com/u/paradb/, allowing users to perform fully functional local installations of ParaDB, in different computational environments, given the platform-agnostic nature of Docker systems (see below).

## Results

### Identification of orthologous genes among the main *Paracoccidioides* isolates

To guarantee consistent genomic annotation of protein-coding genes among *Paracoccidioides* isolates, orthologous genes shared by two or more isolates were initially identified with the aid of the software OrthoFinder [34] and the *Paracoccidioides* spp. pan-genome derived from this analysis, containing all protein-coding sequences within the group, is shown in S3 Fig (a complete description of this pan-genome can be found in the ParaDB website, at http://*paracoccidioides*.com/*paracoccidioides-*orthologous/). Overall, the five isolates display a protein-coding pan-genome composed of 8365 groups of orthologous genes (OG) and share a core genetic pool that consists of 6396 OGs (~75%), reinforcing the close phylogenetic relatedness among members of this group of human pathogenic fungi.

Surprisingly, very few OGs could be found in association with only one isolate. In fact, no exclusive OGs were found in the genomes of Pb3 and Pb300, while Pb18 carries only 2 exclusive OGs of this type (OG0000046 and OG000738) and PbCnh displays only one (OG0006453). Even Pb01, which represents *P*. *lutzii*, the most distantly related species within the *Paracoccidioides* genus, contained only 2 exclusive OGs carrying protein-coding genes (OG0000018 and OG0006445). Not surprisingly, most of these OGs carry genes that typically display structural variations even among closely-related species, since they may perform similar biochemical functions, but interact with alternative substrates: OG0000018 and OG0000046 contain a series of Ser-Thr Protein Kinases, while OG0006445 and OG0006453 contain a series of plasma membrane ATP-binding cassette (ABC) transporters (OG0007385 contain genes that remained identified as hypothetical proteins). Currently, it is not possible to establish whether these genes contribute any kind of adaptive/biological specificities for the different *Paracoccidioides* isolates.

GenBank/RefSeq [20] also contained information regarding a series of non-coding RNAs from the five *Paracoccidioides* spp. isolates, including tRNAs and rRNA genes (18S, 28S and 5S rRNAs) (see Methods). All isolates displayed a similar number of tRNA genes, capable of providing all amino acids required for protein synthesis (see OG0008368 to OG0008478, at http://*paracoccidioides*.com/*paracoccidioides*-orthologous/). A similar situation was observed with the 5S rRNA genes (OG0008365), but the 18S and 28S rRNAs were present in significantly different copy numbers across the genomes of each isolate and could not be found in the genome of isolate PbCnh, probably reflecting problems with the currently available genomic assemblies (see OG0008366 and OG0008367, at http://*paracoccidioides*.com/p*aracoccidioides*-orthologous/). A total of 32 additional ncRNA genes, responsible for transcribing small RNAs (sRNAs), small nuclear RNAs (snRNAs, including the spliceosome RNAs), small nucleolar RNAs (snoRNAs), the Telomerase RNA Component (tercRNA) and the Signal Recognition Particle RNA have also been mapped in the genomes of all five isolates, using as reference, a list of ncRNAs identified in the genomes of Pb18 and Pb01, available from the Ensembl Fungi database [21] ftp site. All *Paracoccidioides* spp. isolates share a common set of such elements, whose orthologues were easily identified in all genomes, with the aid of the short read Bwa-MEM aligner, as described in Methods (see OG0008479 to OG0008510, at http://*paracoccidioides*.com/*paracoccidioides*-orthologous/).

### Functional reannotation of CDSs in the main *Paracoccidioides* isolates

**Fig 1:**
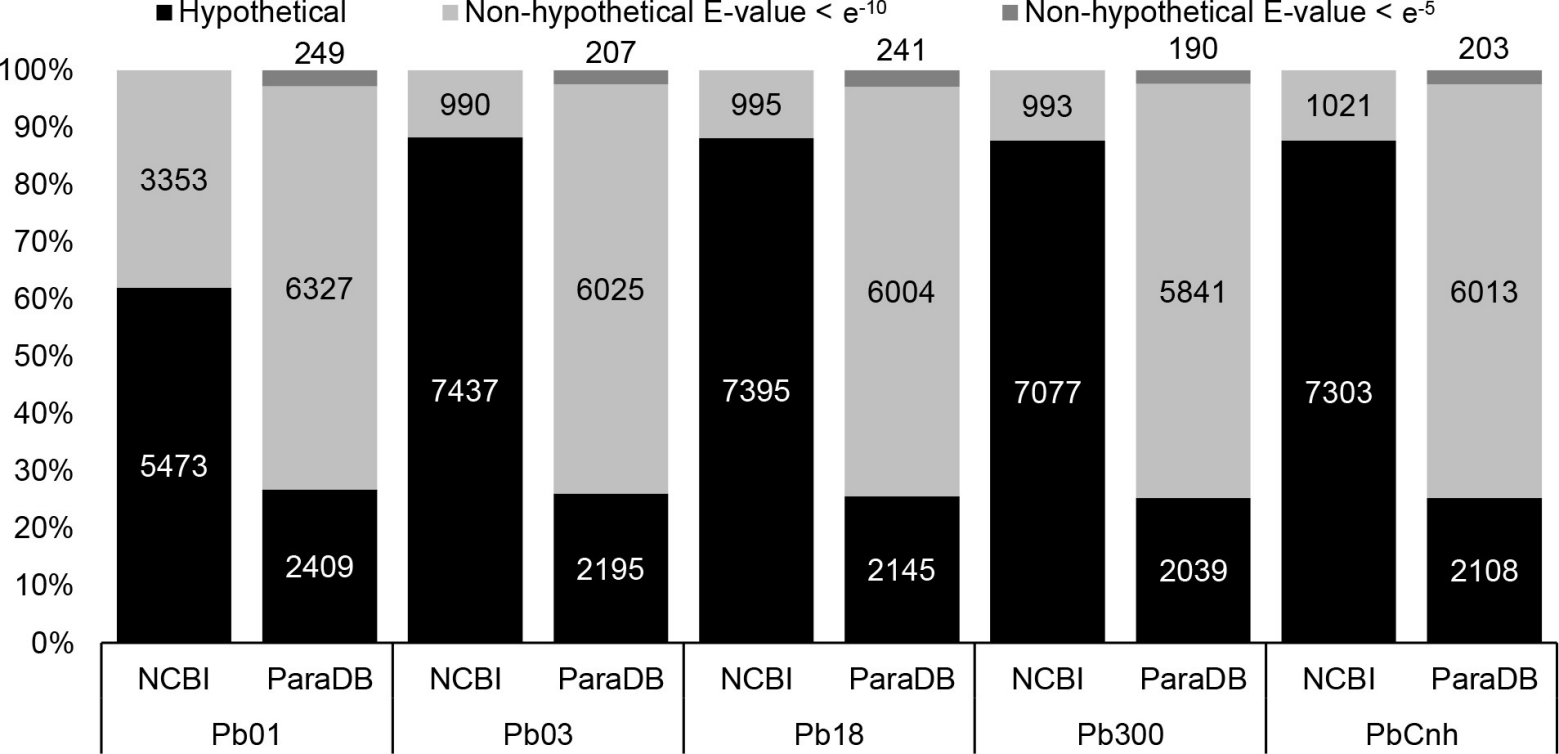
Quantitative assessment of hypothetical and non-hypothetical proteins annotated in the *Paracoccidioides* spp. genomes. The graph indicates the percentage of hypothetical and non-hypothetical proteins present in the ParaDB annotation, when compared to the official annotations available through GenBank/RefSeq, showing that the proportion of CDSs annotated solely as hypothetical proteins was reduced from ~60–90% to ~25–28%, in all strains. Figure also shows the absolute number of CDSs with functional classification, identified at high stringency (E-value < e^-10^) and low stringency (E-value < e^-5^) BLAST analyses, as well as CDSs that remained identified as hypothetical proteins, in the genomes of each *Paracoccidioides* isolate.

To prevent using different nomenclature while annotating corresponding genes across the *Paracoccidioides* genomes, the reannotation process of protein-coding genes was carried out as described in Methods. Thus, genome reannotation of *Paracoccidioides* spp. was initially performed with Pb18 and the annotation data obtained for all genes in this isolate were propagated to the corresponding genes present in the remaining *Paracoccidioides* spp. genomes, using the list of orthologous genes, described above, as a guide. Next, genes present in the genome of Pb01, which did not contain an orthologue in Pb18, were submitted to the same procedure and the same process was successively repeated with the remaining genes from Pb03, Pb300 and PbCnh, generating consistent annotations for all *Paracoccidioides* spp. genomes. As a result, specific functions and/or structural descriptions could be assigned to 6003 out of 8390 protein coding genes mapped in the genome of Pb18, reducing the proportion of genes described in association with hypothetical proteins to 28,5% (2386 genes) (Fig 1). In a second reannotation step, all CDSs that remained identified as hypothetical proteins were submitted to a new BLAST analysis, using a less stringent cutoff (E-value <e^-5^), allowing functional identification of additional 241 CDSs in Pb18, further reducing the proportion of hypothetical proteins in this isolate to 25.5% (2145 genes) (Fig 1). Reannotation of the remaining *Paracoccidioides* spp. genomes provided similar results with the other isolates, reducing the number of CDSs associated with hypothetical proteins to ~29–25% (with E-values < e^-10^ and < e^-5^, respectively), thus increasing the proportion of genes with functional/structural identification to ~71–75% (with E-values < e^-10^ and < e^-5^, respectively) in all cases, significantly improving the annotations of *Paracoccidioides* genomes, in comparison to the annotations currently available in any public biological data repository (see Fig 1).

### Organization of the ParaDB database

Results from the functional reannotations shown in Fig 1 were compiled in ParaDB, a relational database developed to centralize standardized functional genomic annotation from all five reference isolates of the genus *Paracoccidioides*. ParaDB (available at http://*paracoccidioides*.com) presents a simple and intuitive interface, through which such information is made available (S4 Fig). Initial access to the annotation data can be made by the “Databases” button at the center of the webpage, or through a specific pull-down menu, available at the upper right corner of the main ParaDB webpage (S4A Fig). The “Full Database” option directs users to specific annotation data for each of the *Paracoccidioides* isolates under study (S4A Fig). In the “Full Database” mode (S4B Fig), users have initial access to a table that displays each *Paracoccidioides* spp. CDS (identified by numeric codes that correspond to their original GenBank/RefSeq IDs) and their respective ParaDB consensus functional/structural designation. Genes responsible for transcribing ncRNAs, which were not present in the GenBank/Refseq CDS files can also be accessed from these table and carry the suffix NC (non-coding) in the gene IDs assigned to them during our reannotation effort (see Methods). Information regarding data derived from all databases employed in the comparative analyses described above can be accessed by clicking on the (+) symbol, available in each of the CDS cells. Alternatively, such information can be accessed by clicking the “Columns” button on the upper right corner of the table and selecting the desired databases (S4B Fig). In either case, links are provided to direct users to the orthologous genes found in the fungal-specific databases (SGD, CGD and AspGD), where a variety of additional information can be found.

Users may also download the entire annotation files using the “Downloads” button, available from the pull-down menu (S4 Fig). The download site also allows users to access multi-FASTA files (containing either nucleotide or amino acid sequences) for each *Paracoccidioides* strains and/or their respective General Feature (GFF3) format files, which may be of great assistance for a series of downstream analyses, such as the evaluation of data derived from large-scale gene expression experiments involving microarray hybridization, or RNA-seq, for example.

### Local installation of ParaDB using Docker

ParaDB can be accessed and browsed directly on the web, at the URL http://*paracoccidioides*.com, through a variety of platforms, including personal computers of any kind, as well as mobile devices, such as cell phones and tablets, operating under either android or iOS operating systems. However, it is also possible for users to download and install a fully functional version of the database in their own personal computers, avoiding problems derived from low internet trafficking, host server instability, or communication restrictions, due to the presence of firewalls in local servers, for example. To accomplish that, ParaDB was developed in Docker [33], allowing configuration of the entire ParaDB environment in a Docker service, which has been submitted to both GitHub (https://github.com/paracoccidioidesdb) and Open Science Framework (https://osf.io/3sq97/) repositories, ensuring long-term data availability to researchers. This service can be downloaded and used to perform local installations of the database in alternative computing ecosystems, allowing users to conduct their data mining and analyses in a personal and stable working environment. Once installed in a local host, ParaDB will operate from two single containers, called ParaDB-Web and ParaDB-BLAST. Both containers can be consistently interchanged and deployed across different platforms, regardless of hardware and/or operating system (OS) specificities. This implementation is designed to ensure continued and full availability of ParaDB, independently of the original installation in our servers, allowing users to maintain mirrors of the entire database in their local environments. Additionally, the container concept allows the ParaDB infrastructure to be easily scalable, ensuring that hardware resources are provisioned whenever the computational environment reaches its limitations.

Hardware requirements for a local installation of ParaDB are reduced, and a personal computer (or notebook), running on Linux (recommended), containing 2 GB of RAM and 5 GB of available disk space can be used as host for installing a local version of ParaDB. The installation process is extremely simple and only requires previous installations of Docker [33] and Docker Compose in the host machine [48]. Once these components are available, only two steps are required to start a ParaDB environment in the host. In the first step, a Docker-Compose file is downloaded from the GitHub servers to the host machine. In the second step, the Docker Compose is executed, downloading and installing the images/containers of the standard ParaDB modules, starting the service. When Linux is the host machine's operating system, the following commands must be run on the terminal:

$ git clone https://github.com/*Paracoccidioides*DB/paradb.git

$ cd paradb

$ docker-compose up -d

During the deployment process, some ports and disk volumes will be automatically configured on the host machine. Details about the ports and volumes created are available on the ParaDB website. In a standard implementation, ParaDB will use the local host address (IP: 0.0.0.0; HOST: http://localhost) as the default address for internal links. A video demonstrating the entire ParaDB implementation process in a local Linux environment is available at the URL http://paracoccidioides.com/local-install/.

Finally, it is worth mentioning that the ParaDB infrastructure can also be used by independent researchers to develop genome annotation projects involving other closely related fungi and its source code is freely available at: https://cloud.docker.com/u/paradb/.

## Discussion

Databanks dedicated to the storage of genomic data from *Paracoccidioides* spp. started to be designed since the pioneering EST analyses conducted with isolate Pb18 [12,13] and the information provided by these datasets greatly assisted the scientific community in a large number of projects, which contributed to improve our knowledge about this important group of human pathogenic fungi (recently reviewed by [49,50]). However, these original EST databases were gradually abandoned or deactivated after the release of the first draft genomes obtained for isolates Pb18, Pb03 and Pb01 [17]. At this time, data regarding these draft genomes were deposited in a database dedicated to members of the genus *Paracoccidioides*, as part of the Fungal Genome Initiative (FGI), developed by the Broad Institute of Harvard University and the Massachusetts Institute of Technology. This centralized database became the major source of information for subsequent genomic work on *Paracoccidioides* spp., as it contained a great deal of genomic data from these three isolates (including gene functional annotations, chromosome locations of loci and comparative evolutionary analyses of multiple gene sets), as well as tools for searching and downloading genetic sequences and other additional information. However, maintenance of the FGI databases, as well as their respective web interfaces, was discontinued in 2015 and, up to this moment, no other database has been able to reproduce a centralized and efficient environment for genomic analysis on *Paracoccidioides* spp.

Efforts were initially made to incorporate *Paracoccidioides* genomic data into other sites that support comparative analysis of fungal genomes, including MycoCosm and FungiDB [22,23]. FungiDB is a subsection of the EuPathDB family of databases, maintained by the Welcome Trust and NIH. It was designed to combine and make available a plethora of biological information, obtained from a wide variety of microbial eukaryotes. FungiDB [23] includes data from both pathogenic and non-pathogenic fungi and provides information about multiple genomes and gene records, which can be compared and downloaded with the aid of user-friendly browsers. It also integrates genomic data with comments and supporting evidence from the scientific community (including PubMed IDs, images, phenotypic information, etc.) and offers tools for integrating and mining diverse Omics datasets. MycoCosm (supported by JGI/DOE) offers a large collection of fungal genomes, along with interesting web-based tools for alternative types of genome-scale analyses [22].

However, in spite of the effective resources made available through these repositories, the genomic data regarding *Paracoccidioides* isolates that can be currently found in these databases are discrepant, apparently due to absence of manual curation and/or to the confusion generated by the publication of a second version of *Paracoccidioides* genomes (and their respective annotations) [18,19]. For example, detailed analysis of the information available from MycoCosm [22] shows genomic data only for isolates Pb18 and Pb03. However, Pb18 data refer to version 2 of the genome [18], while Pb03 data refer to version 1 [17]. This represents a serious problem for studies involving Pb03, since annotations referring to genomes v1 and v2 display only a portion of common genes [18]. Additionally, the Pb18 genome has ~68% of its CDSs described solely as responsible for encoding hypothetical proteins. EuPathDB/FungiDB [23], on the other hand, presents data for the three *Paracoccidioides* isolates, but only contemplate the genomic information described by [17]. Moreover, their annotations display puzzling results, since the genome of Pb01 displays ~62% of its CDSs identified as hypothetical proteins, while this proportion increases to ~87–88% in the genomes of Pb18 and Pb03. These results represent an unexpected discrepancy, especially when confronted with the comparative analysis of orthologues described herein and shown in S2 Fig. Actually, a closer analysis shows that the data contained in FungiDB [23] appear to have been incorporated directly from NCBI (GenBank/RefSeq) [20] or Ensembl [21], without receiving any manual curation to enhance their accuracy. GenBank/RefSeq and Ensembl are large databases dedicated to providing genomic information for a large number of organisms, relying mostly on automated pipelines to perform genomic annotations. However, these automated pipelines perform comparisons against a large number of independent databanks, resulting in large amounts of data, which are often too complex to be automatically summarized by computer algorithms, requiring manual evaluation by expert reviewers, in order to establish a consensus nomenclature for each analyzed sequence and ensure greater efficiency in the functional identification of the genes present in an organism [51,52,53,54]. Unfortunately, manual curation of genomic data is a time-consuming process and the large number of genomes currently deposited in generic databases, such as GenBank/RefSeq [20] and Ensembl [21], causes many of them to be displayed solely as the result of automated annotation pipelines [55,56].

As expected, manual curation of the *Paracoccidioides* genomes, as shown herein, reduced the proportion of genes described in association with hypothetical proteins from ~90%, in most isolates, to < 30%, in all organisms under study. Moreover, the information regarding these newly annotated genomes have been standardized and made available through a single public database, centralizing the genomic data for the main representatives of the group. Similar improvement in genomic annotation has also been verified with the human pathogenic fungus *Candida albicans*, whose genome has been submitted to manual curation. In fact, the *C*. *albicans* reference genome (RefSeq #GCF_000182965.3) displays ~39% of its genes annotated as responsible for hypothetical proteins, while a manually-curated reannotation, made available through the *Candida* Genome Database (CGD) project [28] reduced the proportion of hypothetical proteins to only ~21% [57].

Unfortunately, microorganisms responsible for neglected diseases tend to attract less attention from the scientific community and, as a result, their genomic annotations are often described with considerably lower accuracy, as manually curated reannotations are rarely performed. For example, the genome of the fungus *Blastomyces dermatitidis*, strain ER-3 (etiologic agent of blastomycosis), available through GenBank (accession #GCA_000003525.2), shows ~ 50% of its genes described in association with hypothetical proteins. Similar scenarios can be verified with the genomes of *Cryptococcus neoformans* var. grubii H99 and *Coccidioides immitis* RS, responsible for cryptococcosis and coccidioidomycosis, respectively, which present 47–49% of their CDSs described in association with hypothetical proteins (see RefSeq accessions #GCF_000149245.1 and GCF_000149335.2). A natural consequence derived from such lack of accuracy is that it is often difficult to use the information derived from these genomic data in large-scale post-genomic studies, such as *in silico* functional/metabolic reconstructions and transcriptome/proteome analyses, which could greatly contribute to improve our knowledge regarding the general biology of these fungi, or the molecular basis of their pathogenicity mechanisms. In this sense, the work described in this manuscript provides manually curated and standardized genomic annotations for the main representatives of all five species of *Paracoccidioides* spp., placing members of this genus in a unique position, when compared with many dimorphic fungi, responsible for neglected mycoses. These new annotations greatly reduce the number of genes identified solely as hypothetical proteins and are integrated into a dedicated database that provides different search/analyses tools to facilitate the development of future post-genomic studies with this important group of human pathogenic fungi.

It must also be highlighted that the data available from ParaDB should provide adequate genomic coverage of protein-coding genes to support *in silico* metabolic analyses in *Paracoccidioides* spp., since the current genomic assemblies display sizes between 29 and 32 Mb, encoding approximately 8000 to 9000 proteins. Thus, gene density in these genomes is 1 CDS/~3.5 kb, which is close to the values observed in well-annotated fungal genomes, such as the cases of *Aspergillus* spp. (1 CDS/~3.1 kb) [29] and *Candida* spp. (1 CDS/~2.3 kb) [28]. Thus, the current *Paracoccidioides* annotations are likely to have identified most (if not all) protein-coding genes present in these fungi, especially when compared with other neglected fungi, such as *H*. *capsulatum* (1 CDS/~4 kb) [58] and *B*. *dermatitidis* (1 CDS/~5.7 kb) [59]. However, information regarding the presence of non-coding elements in *Paracoccidioides* spp. is still scarce and such elements have only recently begun to be unraveled [60]. We expect further versions of ParaDB to incorporate more information on these elements, which can be more efficiently characterized through the analysis of transcriptome data.

Finally, the work presented in this manuscript proposes a pioneering and effective alternative to ensure that the data and resources provided by ParaDB shall remain available in a continued and reproducible way, by providing users with the possibility of installing fully functional mirrors of the database in their own working environments. This was accomplished by developing the entire ParaDB environment in Docker, which allowed the creation of a ParaDB Docker service that was deposited in both GitHub https://github.com/paracoccidioidesdb [61] and Open Science Framework repositories (https://osf.io/3sq97/), two of the world´s largest web-based hosting servers for open source software. The ParaDB image can be freely downloaded and deployed in any kind of local computer, with little infrastructure requirements. The Docker project is providing a new and promising virtualization strategy that consumes a considerably low amount of disk space (when compared to Virtual Machines) and offers the advantage of being platform-agnostic, since it relies on the configuration of containers, which can be consistently interchanged and deployed on different computing environments, regardless the specificities of their hardware and/or operating system [33]. In recent years, this type of technology has been increasingly employed to generate bioinformatics-related software and services to a large variety of research facilities, employing the concepts of *Platform as a Service* (*PaaS*) and *Software as a Service (SaaS)*, as a strategy to assist in replicability and reproducibility of data analysis across laboratories [62,63,64,65,66,67,68]. In this context, ParaDB is the first initiative that tries to develop a Docker system with a biological database, carrying genomic information of pathogenic microorganisms, thus introducing the concept of *Database-as-a-Service* (*DBaaS*), as a strategy to guarantee long term availability of biological data and resources.

## Supporting information

S1 Fig Schematic representation of the reannotation process in *Paracoccidioides* spp. All CDSs from Pb18 were individually BLASTed against INTERPRO, PFAM, Swiss-Prot, SGD (*Saccharomyces* Genome Database), CGD (*Candida* Genome Database) and AspGD (*Aspergillus* Genome Database). Information derived from all these BLAST analyses were compiled in a spreadsheet, along with Gene Ontology (GO) data obtained from DAVID, and such metadata was used to determine a consensus annotation term (ParaDB Annotation) for each CDS (see Methods for details). The information obtained for CDSs from Pb18 were transferred to their respective orthologues, present in the other *Paracoccidioides* isolates, using the list of orthologous genes shown in S3 Fig. as a guide. Next, all CDSs present in the genome of Pb01, which did not contain an orthologue in Pb18, were submitted to the same analysis procedure. The same process was successively repeated with the remaining CDSs from Pb03, Pb300 and PbCnh, generating thorough and consistent annotations for all *Paracoccidioides* genomes.
(TIF)

S2 Fig Schematic representation of the ParaDB computing environment. The ParaDB computing environment was configured in Rancher, as a service/stack, in a Kubernetes cluster, composed of three nodes (one Master and two Worker Nodes). The Master Node contains 2 vCPUs, with 4GB of RAM and 50 GB of disk space, while each Worker Node consists of 4 vCPUs, with 6 GB of RAM and 50 GB of disk space. The system also has 4 GB of RAM and 100 GB of disk space to be used as a buffer, so computational resources can be increased upon demand. The Master Node is responsible for managing the Kubernetes cluster and the Rancher management panel. Worker Node 1 hosts four vCPUs, running independent replicas of the ParaDB-Web Docker Container, which allows web-based access to the main database (including all the software, libraries, dependencies and data necessary to install/run/access MySQL, PHP, Wordpress, and the ParaDB annotations). Worker Node 2 also hosts four vCPUs, running independent replicas of the ParaDB-BLAST Docker Container [containing all the software, libraries, dependencies and data necessary to install/run SequenceServer (https://www.sequenceserver.com/) and the BLAST databases], allowing use of the ParaDB BLAST tool. The redundant implementation of ParaDB-Web and ParaDB-BLAST containers was designed as a warranty to prevent system fail-over. Moreover, the computational environment allows fast provisioning of new replicas of such containers, increasing computational power of the cluster, in case of intensive use. Finally, a monitoring service has also been configured, providing automatic alerts to our team, whenever the operational status of the ParaDB computational environment is compromised (such status can also be checked by users, at http://*paracoccidioides*.com/monitor/).
(TIF)

S3 Fig Pan-genome of *Paracoccidioides* spp. Venn diagram showing the distribution of the 8365 groups of protein-coding orthologous genes (GOs) identified in the genomes of the five *Paracoccidioides* isolates studied herein. Numbers within each area of the Venn diagram correspond to the number of orthologues shared among the five *Paracoccidioides* isolates. A complete list of genes, showing their respective distribution across all *Paracoccidioides* isolates can be found at http://*paracoccidioides*.com/*paracoccidioides*-orthologous/.
(TIF)

S4 Fig Features of the ParaDB user interface. Panel A shows the main page of ParaDB, which allows users to access the annotation data for any of the *Paracoccidioides* spp. genomes, which can be achieved by clicking the “Databases” button at the center of the webpage, or through the pull-down menu, available at the upper right corner of the page. Panel B displays the “Full Database” mode for isolate Pb01, displaying annotation data for each CDS (identified by numeric codes that correspond to their original GenBank/RefSeq annotations) and their respective ParaDB consensus functional/structural designation. Information regarding data derived from all databases employed in the comparative analyses can be accessed by clicking on the (+) symbol, available in each of the CDS cells. Alternatively, such information can be accessed by clicking the “Columns” button on the upper right corner of the table and selecting the desired databases. In either case, links are provided to direct users to the orthologous genes found in the fungal-specific databases (SGD, CGD and AspGD), where a variety of additional information can be found. Keyword-based searches can be made with the aid of the”search” command, shown at the upper right corner of the table (to search all databases at once), or by using the “search filters”, located on the left side of the screen (to limit searches to one of more databases).
(TIF)
