# Supplementary figures and images for: ParaDB: A manually curated database containing genomic annotation for the human pathogenic fungi Paracoccidioides spp
Source: PLoS Negl Trop Dis. 2019 Jul 15;13(7):e0007576. doi: 10.1371/journal.pntd.0007576 (PMC6658007; doi:10.1371/journal.pntd.0007576)

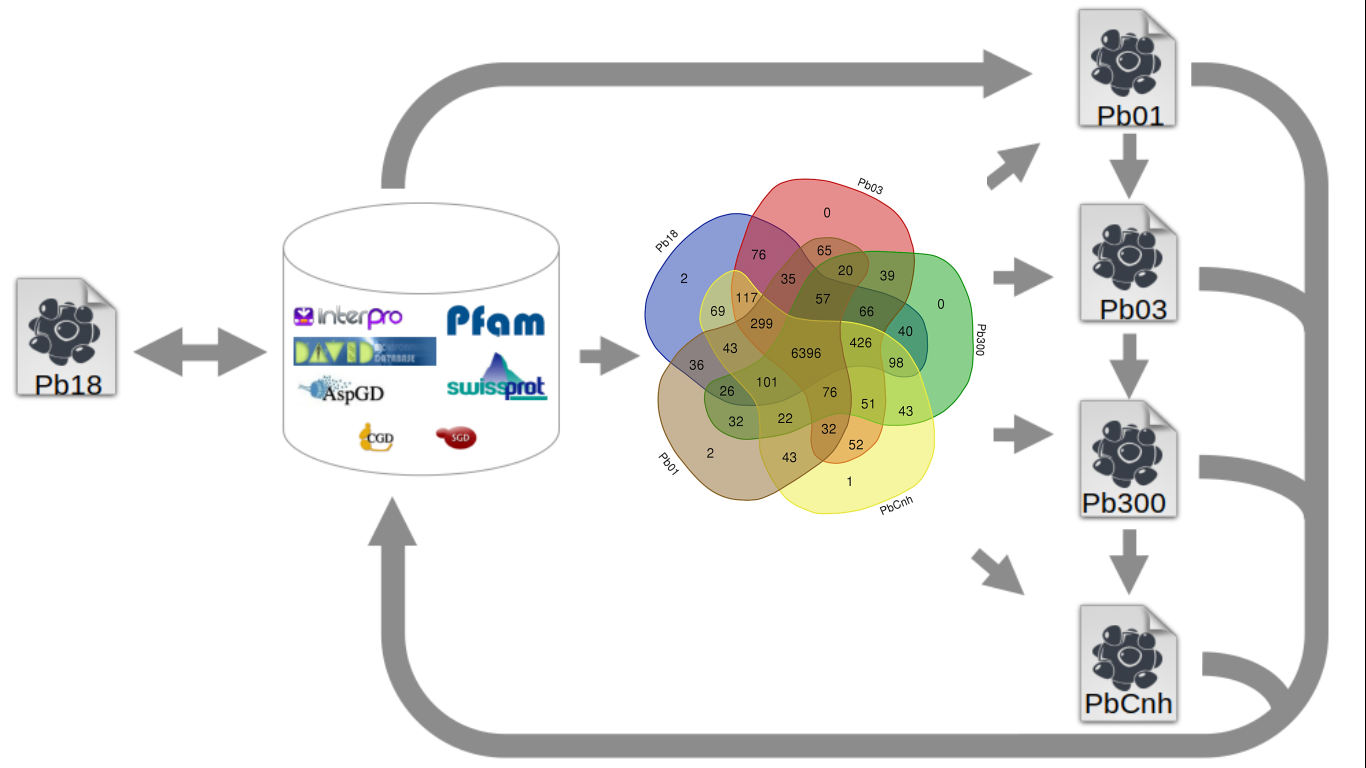

Supplement: S1 Fig — All CDSs from Pb18 were individually BLASTed against INTERPRO, PFAM, Swiss-Prot, SGD (Saccharomyces Genome Database), CGD (Candida Genome Database) and AspGD (Aspergillus Genome Database). Information derived from all these BLAST analyses were compiled in a spreadsheet, along with Gene Ontology (GO) data obtained from DAVID, and such metadata was used to determine a consensus annotation term (ParaDB Annotation) for each CDS (see Methods for details). The information obtained for CDSs from Pb18 were transferred to their respective orthologues, present in the other Paracoccidioides isolates, using the list of orthologous genes shown in S3 Fig. as a guide. Next, all CDSs present in the genome of Pb01, which did not contain an orthologue in Pb18, were submitted to the same analysis procedure. The same process was successively repeated with the remaining CDSs from Pb03, Pb300 and PbCnh, generating thorough and consistent annotations for all Paracoccidioides genomes. (TIF) [file pntd.0007576.s001.tif]

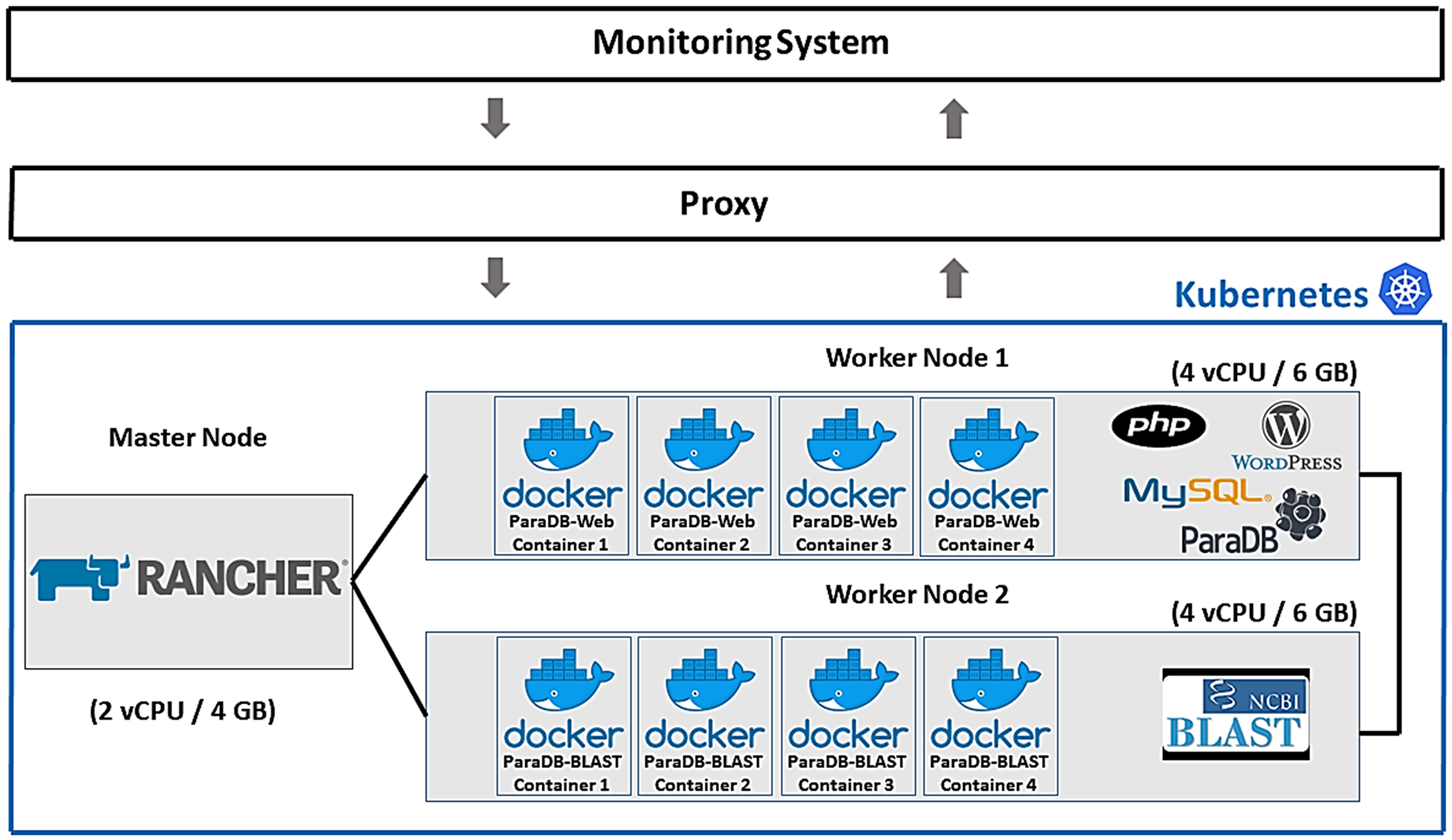

Supplement: S2 Fig — The ParaDB computing environment was configured in Rancher, as a service/stack, in a Kubernetes cluster, composed of three nodes (one Master and two Worker Nodes). The Master Node contains 2 vCPUs, with 4GB of RAM and 50 GB of disk space, while each Worker Node consists of 4 vCPUs, with 6 GB of RAM and 50 GB of disk space. The system also has 4 GB of RAM and 100 GB of disk space to be used as a buffer, so computational resources can be increased upon demand. The Master Node is responsible for managing the Kubernetes cluster and the Rancher management panel. Worker Node 1 hosts four vCPUs, running independent replicas of the ParaDB-Web Docker Container, which allows web-based access to the main database (including all the software, libraries, dependencies and data necessary to install/run/access MySQL, PHP, Wordpress, and the ParaDB annotations). Worker Node 2 also hosts four vCPUs, running independent replicas of the ParaDB-BLAST Docker Container [containing all the software, libraries, dependencies and data necessary to install/run SequenceServer (https://www.sequenceserver.com/) and the BLAST databases], allowing use of the ParaDB BLAST tool. The redundant implementation of ParaDB-Web and ParaDB-BLAST containers was designed as a warranty to prevent system fail-over. Moreover, the computational environment allows fast provisioning of new replicas of such containers, increasing computational power of the cluster, in case of intensive use. Finally, a monitoring service has also been configured, providing automatic alerts to our team, whenever the operational status of the ParaDB computational environment is compromised (such status can also be checked by users, at http://paracoccidioides.com/monitor/). (TIF) [file pntd.0007576.s002.tif]

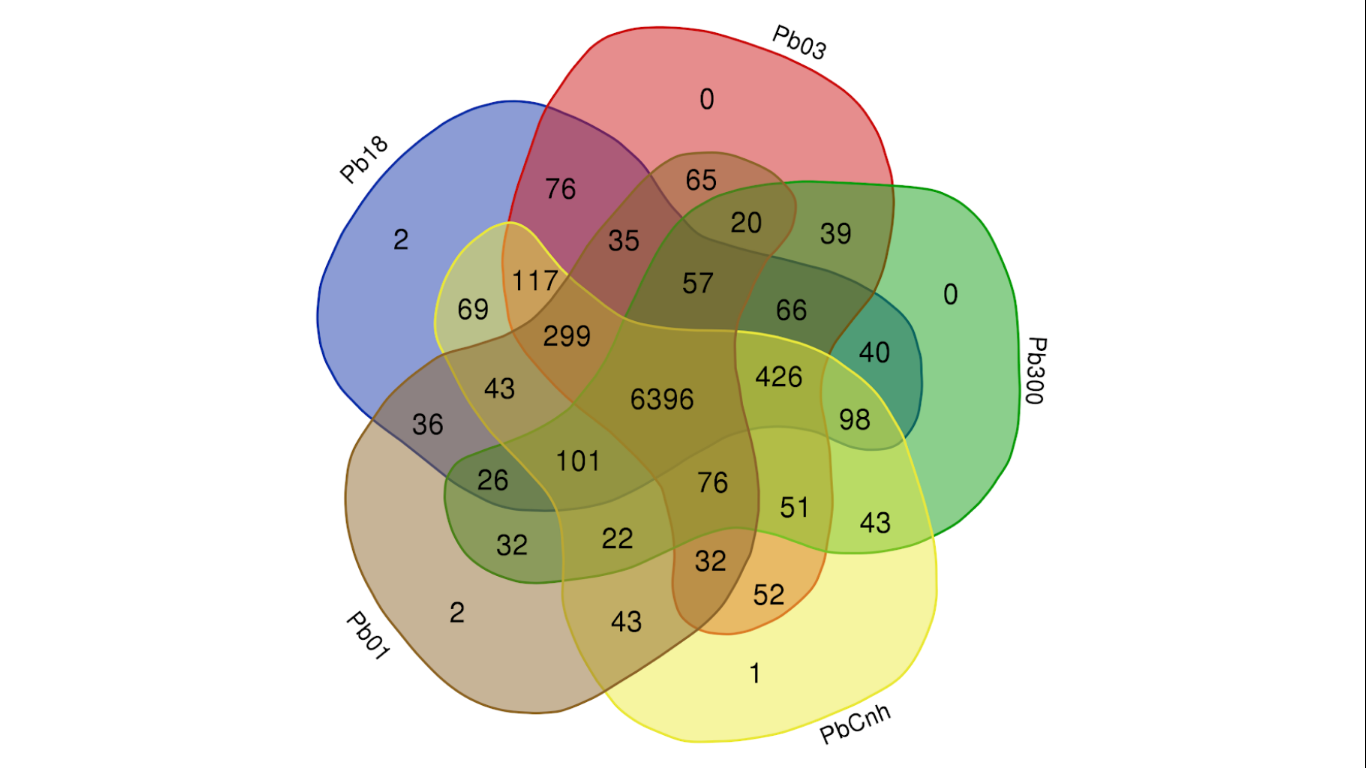

Supplement: S3 Fig — Venn diagram showing the distribution of the 8365 groups of protein-coding orthologous genes (GOs) identified in the genomes of the five Paracoccidioides isolates studied herein. Numbers within each area of the Venn diagram correspond to the number of orthologues shared among the five Paracoccidioides isolates. A complete list of genes, showing their respective distribution across all Paracoccidioides isolates can be found at http://paracoccidioides.com/paracoccidioides-orthologous/. (TIF) [file pntd.0007576.s003.tif]

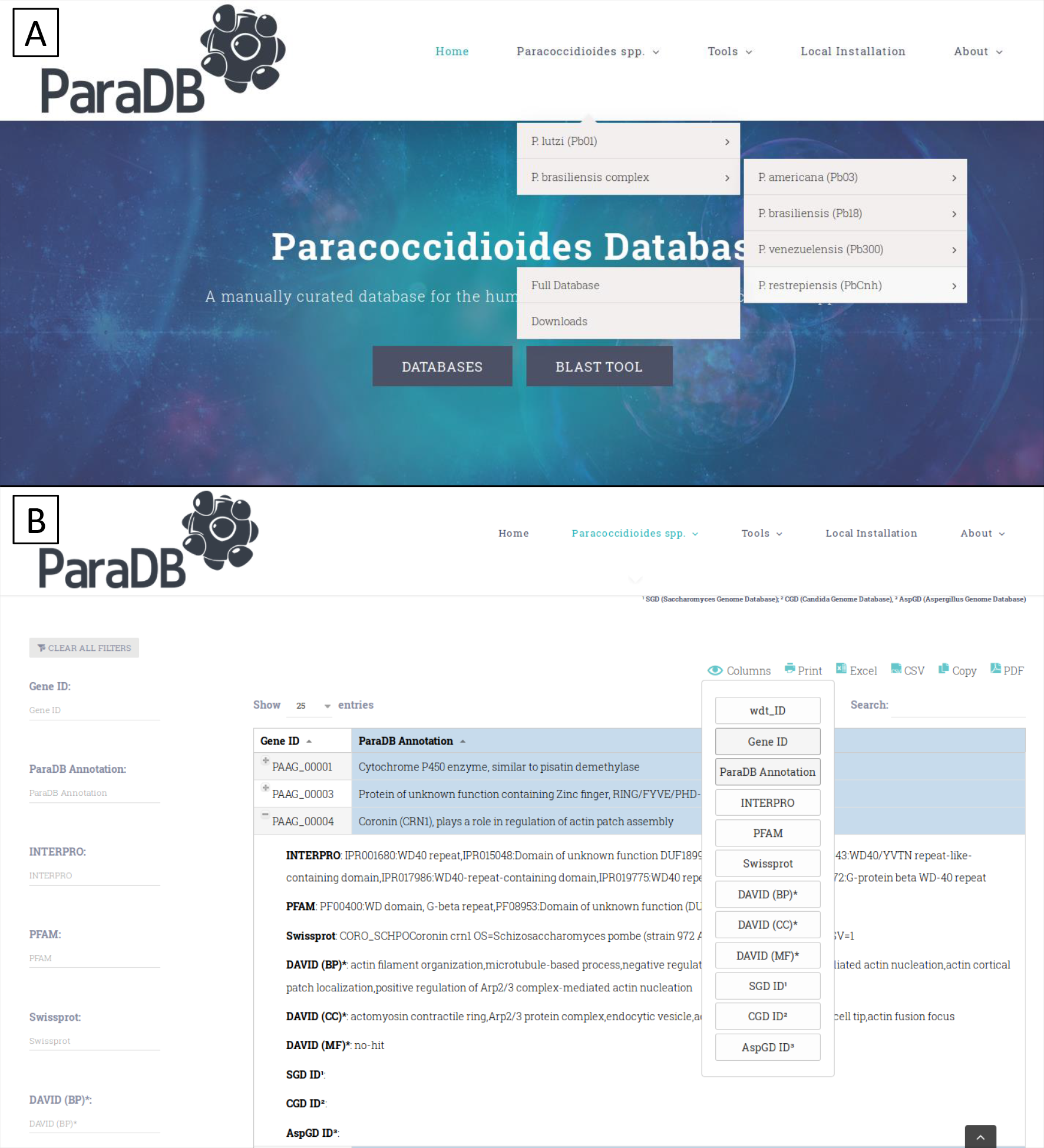

Supplement: S4 Fig — Panel A shows the main page of ParaDB, which allows users to access the annotation data for any of the Paracoccidioides spp. genomes, which can be achieved by clicking the “Databases” button at the center of the webpage, or through the pull-down menu, available at the upper right corner of the page. Panel B displays the “Full Database” mode for isolate Pb01, displaying annotation data for each CDS (identified by numeric codes that correspond to their original GenBank/RefSeq annotations) and their respective ParaDB consensus functional/structural designation. Information regarding data derived from all databases employed in the comparative analyses can be accessed by clicking on the (+) symbol, available in each of the CDS cells. Alternatively, such information can be accessed by clicking the “Columns” button on the upper right corner of the table and selecting the desired databases. In either case, links are provided to direct users to the orthologous genes found in the fungal-specific databases (SGD, CGD and AspGD), where a variety of additional information can be found. Keyword-based searches can be made with the aid of the”search” command, shown at the upper right corner of the table (to search all databases at once), or by using the “search filters”, located on the left side of the screen (to limit searches to one of more databases). (TIF) [file pntd.0007576.s004.tif]
